# Beneficial Effects of Partly Milled Highland Barley on the Prevention of High-Fat Diet-Induced Glycometabolic Disorder and the Modulation of Gut Microbiota in Mice

**DOI:** 10.3390/nu14040762

**Published:** 2022-02-11

**Authors:** Siqi Li, Mengqian Wang, Chang Li, Qingjia Meng, Yantong Meng, Jian Ying, Shuqun Bai, Qun Shen, Yong Xue

**Affiliations:** 1 National Engineering and Technology Research Center for Fruits and Vegetables, College of Food Science and Nutritional Engineering, China Agricultural University, Beijing 100083, China; lisiqi@cau.edu.cn (S.L.); lichang061@sina.com (C.L.); mengyantong@cau.edu.cn (Y.M.); 2 COFCO Nutrition and Health Research Institute Co., Ltd., Beijing 102209, China; wangmengqian@cofco.com (M.W.); mengqingjia@cofco.com (Q.M.); yingjian@cofco.com (J.Y.); 3 National Center of Technology Innovation (Deep Processing of Highland Barley) in Food Industry, China Agricultural University, No. 17 Qinghua East Road, Haidian District, Beijing 100083, China; 4 Key Laboratory of Plant Protein and Grain Processing, College of Food Science and Nutritional Engineering, China Agricultural University, Beijing 100083, China

**Keywords:** highland barley, obesity, insulin resistance, gut microbiota

## Abstract

The nutritional functions of highland barley (HB) are superior to those of regular cereals and have attracted increasing attention in recent years. The objective of this study was to investigate whether partly milled highland barley (PHB) can regulate the serum glucose and lipid disorders of mice fed a high-fat diet (HFD) and to further explore their potential gut microbiota modulatory effect. Our results showed that PHB supplementation significantly reduced fasting blood glucose (FBG) and improved oral glucose tolerance. Histological observations confirmed the ability of PHB to alleviate liver and intestine damage. Furthermore, the results of 16S amplicon sequencing revealed that PHB prevented a HFD-induced gut microbiota dysbiosis, enriching some beneficial bacteria, such as *Lactobacillus*, *Bifidobacterium,* and *Ileibacterium*, and reducing several HFD-dependent taxa (*norank_f_Desulfovibrionaceae*, *Blautia*, *norank_f_Lachnospiracea*e, *unclassified_f_Lachnospiracea*e, and *Colidextribacter*). In addition, the increase of *Lactobacillus* and *Bifidobacterium* presence has a slightly dose-dependent relationship with the amount of the added PHB. Spearman correlation analysis revealed that *Lactobacillus* and *Bifidobacterium* were negatively correlated with the blood glucose level of the oral glucose tolerance test. Overall, our results provide important information about the processing of highland barley to retain its hypoglycemic effect and improve its acceptability and biosafety.

## 1. Introduction

With rapid economic development and the acceleration of industrialization, urbanization, and globalization, human lifestyles, especially diet, have undergone considerable changes which greatly contribute to the increase of metabolic diseases [1]. The Global Burden of Disease Study has shown that metabolic disorder-related factors, such as high fasting plasma glucose, high systolic blood pressure, etc., are the main reasons for the high mortality rate of cardiovascular diseases [2]. Some strong pieces of evidence from both observational and intervention studies have shown that the higher consumption of whole grain is associated with a lower incidence of and mortality from hypertension [3], cardiovascular diseases [4], type 2 diabetes [5,6], and some cancers [7,8].

The nutritional function of highland barley (HB, *Hordeum vulgare L. var. nudum hook. f*) outshines that of ordinary grains, partly due to its high protein [9,10] and high β-glucan [10,11], and is gradually attracting widespread attention [12]. β-glucan derived from HB can inhibit the activities of key enzymes (such as α-glucosidase, α-amylase, and invertase) in glucose metabolism in vitro [13], and the evidence in animal experiments indicates that β-glucan maybe improve cell proliferation by targeting the mammalian target rapamycin (mTOR) and regulating the protein kinase B (Akt)/glycogen synthase kinase-3 beta (GSK-3 beta) pathway, thereby ameliorating β-cell dysfunction, and has a synergistic effect compatible with phenolic substances such as chlorogenic acid and (-)-epicatechin [14,15]. Accumulated research has shown that HB has the potential to prevent or contribute to the treatment of cancer [16], cardiovascular diseases [17], and metabolic syndrome [18,19,20]. In addition, some animal studies demonstrated that intake of whole HB could not only improve glucose and lipid metabolism [19], but also change the structure of the intestinal microbiota [21]. However, most of the current research was based on whole highland barley grain (WHB) which has a rough taste and is difficult to shape during processing [22]. Additionally, WHB is susceptible to microbial and mycotoxin contamination during harvesting, transportation, and storage [23,24]. To solve the taste and biosafety problem of WHB, it may be possible to remove part of the HB bran through a moderate milling treatment. Though it is unknown whether the hypoglycemic and hypolipidemic effects of the milled HB still exist.

Therefore, this study was conducted to assess the ability of partly milled HB to combat metabolic disorders induced by a high-fat diet (HFD). Firstly, the effects of PHB with different doses on glucose and lipid metabolism in mice fed HFD were assessed by observing the changes of physiological indicators such as bodyweight and tissues weight, blood lipid, fasting, and postprandial blood glucose, as well as histopathology. Furthermore, the structure of the mice’s intestinal microbiota was evaluated to discover the underlying mechanism of improved glucose metabolism by using 16S amplicon sequencing. Finally, to explore the associations of physiological indicators with the differential genera, a correlation analysis between physiological indicators and the relative abundance of the individual genus was also conducted.

## 2. Materials and Methods

### 2.1. Materials and Reagents

Partially milled Shigatse highland barley (Zangqing 2000) (PHB, with 10% milling degrees) was kindly provided by COFCO Nutrition and Health Research Institute (Beijing, China). Paraformaldehyde fix solution was purchased from Wuhan Servicebio Technology Co., Ltd. (Wuhan, China). Total cholesterol (TC) and triglyceride (TG) detection kits were purchased from Nanjing Jiancheng Institute of Bioengineering (Jiangsu, China). Other experimental chemical reagents such as glucose used in this study were purchased from Sinopharm Chemical Reagent Co., Ltd. (Beijing, China).

### 2.2. Animals and Diets

Fifty male C57BL/6J mice of specific pathogen-free (SPF) grade (6 weeks old) were purchased from Beijing Vital River Laboratory Animal Technology Co., Ltd. (Beijing, China), and were reared in the SPF animal laboratory at 23 ± 2 °C, relative humidity of 55 ± 5% with a 12 h cycle of light-dark. The animals had ad libitum access to food and water during the experiment. All the procedures were carried out under the permission of the Animal Care Committee of China Agricultural University (AW08012020-4) and performed in strict accordance with the guidelines of the National Research Council Guidelines. After 1 week of acclimation, mice were randomly assigned to five groups (n = 8 per group): (1) a normal-chow (NC) group which was fed a low-fat diet (LFD, 10 kcal% fat, D12450J; Research Diets, New Brunswick, NJ, USA); (2) a model control (MC) group which was fed a high-fat diet (HFD; 60 kcal% fat, D12492; Research Diets, New Brunswick, NJ, USA); (3) a low-dose (LD) group which was fed a HFD containing 10% (*w*/*w*) PHB (Changzhou Shuyishuer Bio-Tec Co., Ltd., Changzhou, China); (4) a middle-dose (MD) group which was fed a HFD containing 20% (*w*/*w*) PHB; and (5) a high-dose (HD) group which was fed a HFD containing 30% (*w*/*w*) PHB. The nutrient contents of all highland barley-supplemented diets were designed with reference to HFD D12492. The detailed compositions and energy densities of the experimental diets are listed in Table S1. All groups were fed for a period of 12 weeks.

The energy intake of the mice was measured twice a week and their bodyweight was recorded weekly. On weeks 0, 6, and 12, after a 12-h fast, all mice underwent a 2-h oral glucose tolerance test (OGTT). Following oral gavage of D-glucose (2 g/kg body weight), blood samples were collected from a tail vein at 0, 15, 30, 60, 90, and 120 min, glucose levels were determined using a blood glucose meter (Contour TS 1816; Bayer Co., Ltd., Leverkusen, German), and the area under the curve (AUC) was calculated to evaluate the glucose tolerance.

### 2.3. Sample Collection

At the end of the intervention (week 12), fresh stools were collected. At the end of the experiment, mice were anesthetized with chloral hydrate following 12 h of fasting. The serum was separated by centrifugation of whole blood obtained from the internal canthal vein and then stored at −80 °C. After cervical dislocation execution, adipose tissue, ileum, and colon were collected and weighed. Some tissues (liver, ileum near the cecum end, colon near the cecum end) were fixed in 4% paraformaldehyde fix solution and used for pathological sectioning, and the rest were stored at −80 °C.

### 2.4. Biochemical Parameters

The level of TC and TG in serum was measured by an automatic blood biochemistry analyzer (COBAS INTEGRA 800, Roche, Switzerland). Sample preparation and loading procedures were carried out in accordance with standard operating procedures.

### 2.5. Histological Analysis

After being fixed in 4% paraformaldehyde (*v*/*v*), the tissues, including the liver, ileum near the cecum end, and colon near the cecum end were dehydrated using a series of ethanol solutions. Following treatment with xylene to make them clear, tissues were immersed in paraffin at 65 °C and then embedded in paraffin. Sections 5 μm in size were cut using a microtome (RM2016, Shanghai Leika Instruments Ltd., Shanghai, China) and stained with hematoxylin and eosin (H&E), then oven dried at 60 °C for 1 h. Stained paraffin sections were sealed with neutral gum and images were viewed using a computer-integrated microscope (BX51, Olympus Corporation, Tokyo, Japan).

### 2.6. Gut Microbiota Analysis

Gut microbiota composition was determined using high throughput 16S rRNA amplicon sequencing. The total DNA was extracted from stool samples using the E.Z.N.A.Ò soil kit (Omega Bio-tek, Norcross, GA, USA) according to the manual. The DNA concentration and purity were measured using a NanoDrop2000 spectrophotometer (Thermo Fisher Scientific, Waltham, MA, USA) and the DNA extraction quality was measured using 1% agarose gel electrophoresis. PCR targeting the V3-V4 region of the 16S rRNA gene was done with primers 338F {5′-ACTCCTACGGGAGGCAGCAG-3′} and 806R {5′-GGACTACHVGGGTWTCTAAT-3′}. The PCR product was recovered using 2% agarose gel and the recovered product was purified using AxyPrep DNA Gel Extraction Kit (Axygen Biosciences, Union City, CA, USA) and quantified using Quantus™ Fluorometer (Progema Corporation, Madison, WI, USA). Following construction of an amplicon library using the TruSeq^TM^ DNA Sample Prep Kit (Illumina, San Diego, CA, USA), sequencing of the purified amplified fragment was performed using the Illumina Miseq PE300 platform at Majorbio Bio-Pharm Technology Co., Ltd. (Shanghai, China).

The raw data from the 16S rRNA gene sequencing were filtered and trimmed and operational taxonomic units (OTUs), clustering of the sequence, and chimera removal were performed using the UPARSE software (http://drive5.com/uparse/, version 7.0.1090 (accessed on November 2021)) based on 97% similarity. The classification sequence was annotated using RDP Classifier (http://sourceforge.net/projects/rdp-classifier/, version 2.11 (accessed on November 2021)) by comparing to the Silva database (http://www.arb-silva.de (accessed on November 2021), version 138) with a threshold of 70%. R Stats Software version 3.3.1 (R Core Team, Vienna, Austria) was used for statistics and graphing. The alpha-diversity index of bacterial communities was tested by Wilcoxon rank-sum on the OTU level and the β-diversity was analyzed by principal coordinates analysis (PCoA) on a genus level based on Bray-Curtis distance.

### 2.7. Statistical Analysis

Data were analyzed using SPSS version 20.0 (IBM SPSS Inc., Armonk, NY, USA) and R Stats Software (R Core Team, Vienna, Austria). Charts were drawn using GraphPad Prism version 9.0 (GraphPad Software, San Diego, CA, USA) and R Stats Software. Significant differences between groups were compared by one-way analysis of variance (ANOVA), Wilcoxon rank-sum test, analysis of similarities (ANOSIM), or Kruskal-Wallis H test. Correlation analysis between physiological indicators and the relative abundance of an individual genus was performed using Spearman correlation. All results were expressed as the mean ± SEM or median (range interquartile) and *p* < 0.05 was considered as a significant difference.

## 3. Results

### 3.1. Effects of PHB on the Bodyweight, Food Intake, and Tissues Weight

**Figure 1:**
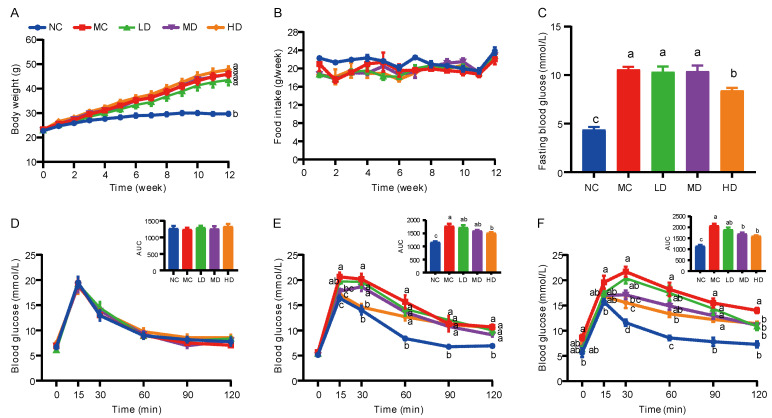
Effect of PHB on the bodyweight, food intake, and glucose metabolism in HFD mice. (**A**) Bodyweight. (**B**) Food intake. (**C**) Fasting blood glucose. (**D**–**F**) OGTT curve and AUC at week 0 (**D**), week 6 (**E**), and week 12 (**F**), respectively. Results are presented as mean ± SEM (*n* = 8). Different letters are significantly different (*p* < 0.05) based on Tukey multiple range test. PHB, partly milled highland barley; NC, normal control group; MC, model control group fed HFD; LD, low-dose group fed HFD containing 10% PHB; MD, middle-dose group fed HFD containing 20% PHB; and HD, high-dose group fed HFD containing 30% PHB.

**Figure 2:**
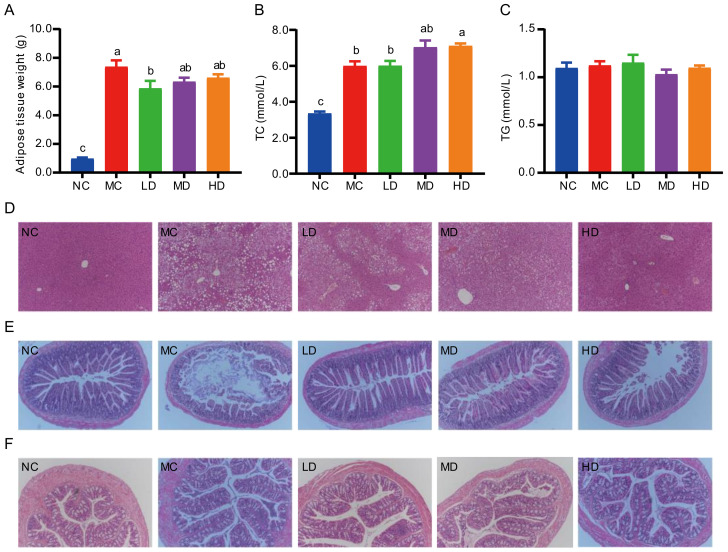
Effect of PHB on tissue weight, serum lipid profiles, and histopathology of liver, ileum, and colon. (**A**) Adipose tissue weight. (**B**) Serum total cholesterol. (**C**) Serum triacylglycerol. (**D**–**F**) Hematoxylin and eosin staining of the liver (**D**), ileum (**E**), and colon (**F**) at 100× magnification, respectively. Data are expressed as mean ± SEM (*n* = 8). Different letters are significantly different (*p* < 0.05) based on Tukey multiple range test.

As shown in Figure 1A, the bodyweight of mice fed with a HFD (MC group) was significantly higher than that of the NC group since week 4. In comparison with the MC group, the bodyweight of the LD group exhibited a slight downward trend. However, there was no significant difference in bodyweight between the intervention groups of PHB and the MC group (Figure 1A). Throughout the feeding period, there were no statistical differences in the food intake (g/week) between the MC group and HB supplemented groups. (Figure 1B). Interestingly, the adipose tissue weight of the LD group was significantly lower than that of the MC group (Figure 2A).

### 3.2. Effects of PHB on Glucose Tolerance

Co-administrated PHB and HFD with three doses (low dose: 10% PHB, LD; middle dose: 20%, MD; and high dose: 30%, HD) were utilized to investigate the effects of PHB on the glucose metabolism of mice fed with a HFD. The results showed that the MC group (10.6 ± 0.27 mmol/L) displayed a significantly higher FBG level than the NC group (4.4 ± 0.29 mmol/L) after a 12-week intervention (Figure 1C). In comparison with the MC group, the FBG level showed a downward trend after PHB supplementation, although it was not significant except for the HD group (8.4 ± 0.29 mmol/L, *p* < 0.01). 

OGTT was conducted at 0, 6, and 12 weeks to evaluate the ability of mice to regulate blood glucose and the function of pancreatic β-cells. From Figure 1D, it can be seen that at week 0, there was no significant difference in the 2-h glucose tolerance curve and the AUC of the mice fed different diets (*p* > 0.05), showing that the blood glucose of the control group was the same as that of all groups in the initial state. The OGTT results showed that after 6 weeks of intervention, the blood glucose level of the MC group was significantly higher than that of the NC group at all time points except the starting point (Figure 1E, *p* < 0.001) and the AUC indicated that the MC group had severely impaired glucose tolerance (*p* < 0.001). However, the HD group exhibited significantly lower blood glucose levels at 15 and 30 min after oral administration of the glucose solution (*p* < 0.01) and its AUC was also significantly reduced (*p* < 0.05). After being supplemented with PHB for 12 weeks, as the addition of PHB increased, the AUC of each intervention group gradually decreased (Figure 1F). Among them, the MD (*p* < 0.05) and HD groups (*p* < 0.01) were significantly lower than the MC group.

### 3.3. Effects of PHB Supplementation on Triglyceride and Total Cholesterol Levels in the Serum

Serum lipid profiles were detected to explore the hypolipidemic effects of PHB on high-fat-fed mice. In comparison with the NC group, HFD feeding resulted in a significant increase in the level of TC but not TG at the end of the intervention (Figure 2B,C, *p* < 0.05). PHB intervention did not have a good effect on reducing the TC level of HFD fed mice. On the contrary, the TC level of the HD group was significantly higher than that of the MC group (*p* < 0.05).

### 3.4. Effects of PHB on Histopathological Alterations of Liver Tissues

To further explore the liver function, a histological examination of the livers was conducted by H&E staining. As shown in Figure 2D, the liver cells in the NC group were of normal morphology, neatly arranged, and had obvious nuclei in the center. In contrast, the liver tissues of mice in the MC group suffered severe liver damage under the induction of the HFD. Liver cells were filled with lipid vacuoles and the nucleus was squeezed to one side. However, after 12 weeks of intervention with PHB, especially the high dose, the hepatic histopathological damages of the mice fed HFD were ameliorated with a normal appearance, less vacuolization, and fewer lipid droplets.

### 3.5. Effects of PHB on Histopathological Alterations of Ileum Tissues and Colon Tissues

H&E staining showed the pathological changes of mouse ileum tissues and colon tissues at the end of the intervention. From Figure 2E, it can be seen that the ileum structure was normal and intestinal villi were lined in neat rows in the NC group while the villi were shortened and fell off with local epithelial shedding in the model group. Different degrees of improvement on villi shedding were found in the intervention groups compared with the MC group. Briefly, the mucosal structure of ileum tissues under the PHB intervention was improved, and the intestinal villi were arranged neatly, with only a small amount of inflammatory cell infiltration observed.

Furthermore, the colon tissue structure of the mice in the control group was analyzed with obvious crypts and a large number of goblet cells, regular edges of the crypts, and no abnormal infiltration of inflammatory cells. Compared with the normal group, the MC group showed a mild pro-inflammatory state with irregular crypt edges, a large decrease in goblet cells, and neutrophils infiltrating the mucosal layer and crypts. Low-, medium and high-dose PHB treatment reduced inflammatory reactions. Low- and middle-doses of PHB inhibited inflammation compared with the MC group, whereas the suppression of inflammation achieved by high PHB treatment was similar to that observed in the normal group (Figure 2F).

### 3.6. Effects of PHB on the Diversity of Mice Fecal Microbiome

**Figure 3:**
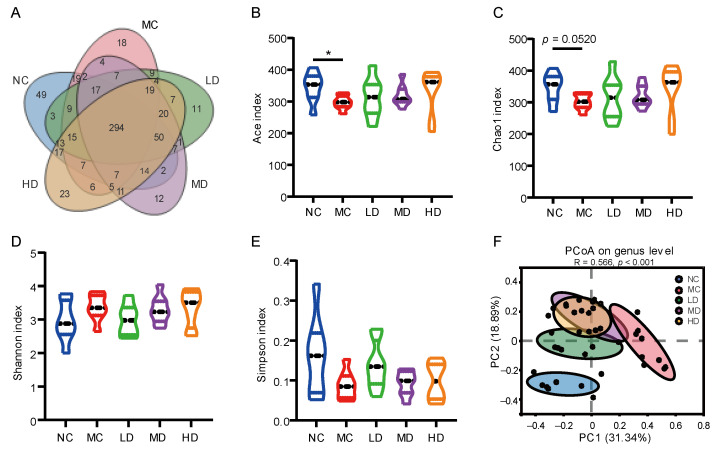
Effect of PHB on the diversity of the fecal microbiome. (**A**) A Venn diagram showing the overlap of the OTUs in the fecal microbiome. (**B**–**E**) Changes in Ace (**B**), Chao 1 (**C**), Shannon (**D**), and Simpson (**E**) alpha diversity index. (**F**) Principal coordinates analysis (PCoA) based on the Bray Curtis distance of fecal microbiota. Significance was determined using Wilcoxon rank-sum test; * *p* < 0.05 (*n* = 8).

Characterization of fecal bacterial communities was performed by 16S rRNA gene sequencing. The Venn diagram displayed the common and unique OTUs between groups (Figure 3A). There were fewer unique OTUs in each group and the majority of OTUs were shared by multiple groups. Among them, 294 OTUs were shared by five groups. Alpha-diversity was estimated through the Ace index, Chao1 index, Shannon index, and Simpson index. Figure 3B–E show that the HFD-feeding caused changes in the alpha-diversity of the fecal microbiota. Compared with the NC group, the Ace index of the MC group was significantly lower (*p* < 0.05), but the Chao 1 index (*p* = 0.052), Shannon index (*p* > 0.05), and Simpson index (*p* > 0.05) were not significantly changed. While the effect of a HFD on the microbiota diversity was partly restored by PHB, there was no significant difference between the diversity index of the PHB intervention groups and the NC group (Figure 3B–E). The difference in the fecal microbiota structure among the five groups was visualized using PCoA analysis on the basis of the Bray-Curtis distance and the results are displayed in Figure 3F (PC1 and PC2 were 31.34% and 18.89%, respectively). The NC group and the MC group were substantially separated along the PC1 axis which suggested remarkably different structures of the gut microbial community. In addition, the PHB intervention groups were closer to the NC group than the MC group.

### 3.7. Effects of PHB on the Composition of Mice Fecal Microbiota

**Figure 4:**
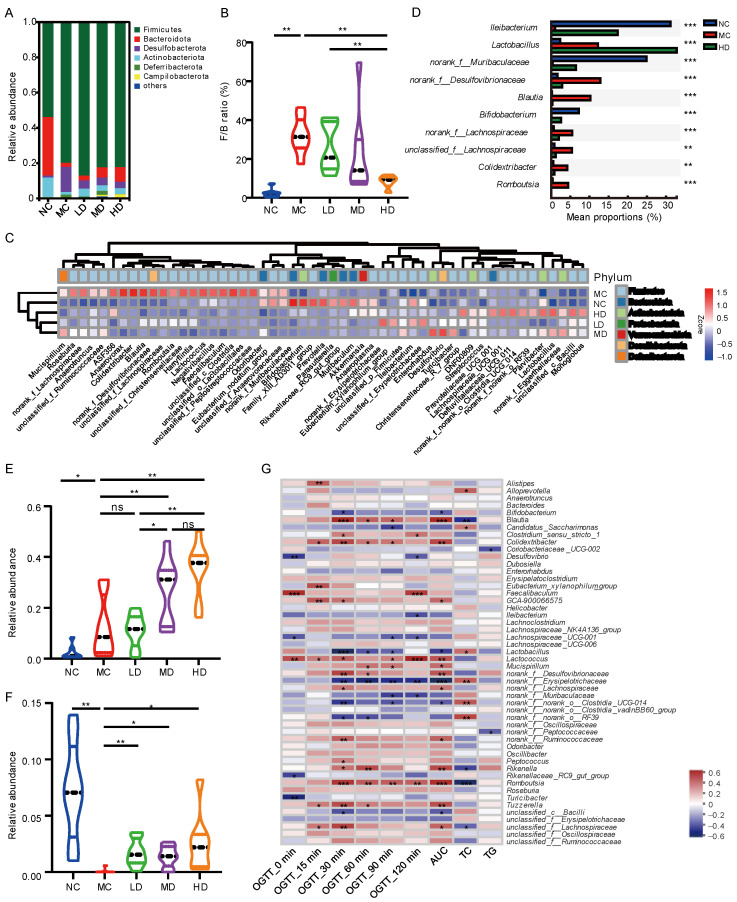
Effect of PHB on the composition of the fecal microbiome. (**A**) fecal microbial composition at the phylum level. (**B**) *Bacteroidetes*/*Firmicutes* ratio (*n* = 6 of HD group). (**C**) Heatmap analysis at the genus level. (**D**) The top 10 species with the most significant differences in relative abundance at genus level between NC group, MC group, and HD group. (E-F) Relative abundance of *Lactobacillus* (**E**) and *Bifidobacterium* (**F**). (**G**) Heatmap of Spearman’s correlation analysis between fecal microbiota and glucose and lipid metabolism-related indexes. Significance was determined using Kruskal-Wallis H test, or Wilcoxon rank-sum test; * *p* <0.05, ** *p* <0.01, *** *p* <0.001 (*n* = 8). OGTT_*t* min, blood glucose level of mice at *t* min after oral administration of glucose after 12 weeks of intervention; AUC, the area under the curve of the 2-h oral glucose tolerance test at 12 weeks; TC, total cholesterol; TG, triglyceride.

To assess the role of PHB in gut microbiota, the composition of the bacteria was analyzed. Figure 4A shows that *Firmicutes* and *Bacteroidetes* were the dominant bacteria in mice feces at the phylum level. The HFD resulted in higher *Firmicutes* and lower *Bacteroidetes*; such change was evidently reversed by PHB supplementation (Figure 4A). Similarly, the *Firmicutes*/*Bacteroidetes* (F/B) ratio of the MC group was significantly higher than that of the NC group (*p* < 0.05) while the HD group exhibited a lower F/B ratio compared with the MC group (Figure 4B; *p* < 0.05). Additionally, a taxonomic heatmap was clustered based on the Bray-Curtis distance to visualize the relative abundance of bacteria with significant differences in abundance in five groups. The dominant bacteria of the HD group were relatively similar to those of the NC group but different from those of the MC group (Figure 4C). The differences in species abundance among the NC group, MC group, and HD group at the genus level were compared through the Kruskal-Wallis H test. The *p*-value was corrected for multiple tests by FDR, and the Tukey-Kramer post-hoc test was performed at a significance level of 0.05. Among the top 10 genera in terms of abundance, *Ileibacterium*, *norank_f__Muribaculaceae*, and *Bifidobacterium* were significantly decreased in the MC group, while *norank_f_Desulfovibrionaceae*, *Blautia*, *norank_f_Lachnospiraceae*, *unclassified_f_Lachnospiraceae*, *Colidextribacter*, and *Romboutsia* were significantly increased (Figure 4D). The enrichment or reduction of the above abnormal bacteria in the HD group was restored. Notably, the relative abundance of *Lactobacillus* was significantly higher in the MC group than that in the NC group (Figure 4E). However, PHB treatment resulted in a significant increase in the relative abundance of *Lactobacillus* than in the MC group which displays a dose-response relationship, though not significant, between some doses. Similarly, there was a dose-response trend between *Bifidobacterium* with PHB, but it is not significant (Figure 4F).

### 3.8. Correlation between Gut Microbiota and Related Indicators of Glucose and Lipid Metabolism

To explore the potential relationships between the gut microbiome changes and physiological indicator changes, a correlation matrix was generated using the Spearman correlation (Figure 4G). The abundance of most genera such as *Lactobacillus*, *Bifidobacterium*, *Ileibacterium*, *Lachnospiraceae_UCG_001*, *norank_f_Erysipelotrichaceae* and *norank_f_Muribaculaceae, norank_f_norank_o_Clostridia_UCG-014* were significantly negatively correlated with the level of postprandial blood glucose and were positively correlated with the level of TC.

## 4. Discussion

The association between the increased intake of whole grains and reduced disease risk, such as cardiovascular diseases [25], type 2 diabetes [26], and hypertension [27] has been well-documented. Many countries recommend increasing the intake of whole grains as a result [28]. However, whole grain foods are facing challenges in terms of sensory properties and biological safety [29]. A survey found that the perception of the sensory properties of whole grain is the most important factor of the main obstacles and promoting factors of whole grain intake [30]. Moderately milling grains can minimize the level of undesirable substances such as bacteria, molds, agrochemicals, and heavy metals and improve the mouthfeel and taste of the grains while maximizing the retention of active ingredients such as polyphenols, dietary fiber, and β-glucan [31]. In order to explore whether the moderately milled grains still have biological effects similar to that of the whole grain mentioned previously, this study took one of the current research hotspots-highland barley [22,32] as the research object. By comparing the nutrient composition and in vitro digestion properties of HB with a 10%, 20%, and 30% milling degree (Table S2–S4), we finally selected highland barley with a 10% milling degree (PHB) for a 12-week intervention experiment in mice fed a HFD. This experiment set up three dose groups (10%, 20%, and 30%). The results indicated that high-dose PHB had a positive effect on improving blood glucose, histopathology, and intestinal flora of HFD mice. A 20% addition of PHB exhibited a significant effect of lowering blood glucose, and the effect was even more notable when the addition amount of PHB was 30%. However, PHB intervention had no significant positive effect on the bodyweight and blood lipids of mice fed a HFD.

The current evidence on the effect of whole grain intake on obesity is inconsistent. The initial epidemiological evidence demonstrated that a higher whole grain intake was associated with weight loss [33,34] while the latest meta-analysis suggested that whole grain intake had no significant effect on bodyweight [35]. Although animal experimental evidence indicated that HB had a weight-reducing effect [19,36] and hypolipidemic effect [37,38], it was observed that high doses of WHB increased the weight of rats in the study by Xia et al. [38]. The difference between these results may be caused by differences in the variety of dosages of HB. There are differences in the content and composition of active ingredients (such as β-glucan, phenolic acid, etc.) in different varieties of barley [39,40,41]. Meanwhile, the milling treatment can result in the loss of dietary fiber and polyphenols (Table S2) which may weaken the anti-obesity and anti-hyperlipidemic ability of HB [12]. 

Diabetes is prevalent worldwide and has become a major disease burden. In recent years, HB has been demonstrated to regulate glucose metabolism and ameliorate hyperglycemia and has been recommended as a suitable food option for people with diabetes problems [19,20,41]. Our study showed that HB after a partly milled treatment still had good hypoglycemic ability (Figure 1C–F). Current research shows phenolic substances (Procyanidin B1, ferulic acid, and *p*-Coumaric Acid) [41,42] and β-glucan [14,15] enriched by HB play a major role in regulating blood glucose. Although the milling process will cause the loss of polyphenols, it will also expose β-glucan (Table S2), which may be the reason why PHB still has the ability to lower blood glucose.

Accumulated data indicates that gut microbes and their metabolites are related to the course, prevention, or treatment of obesity, type 2 diabetes, ulcerative colitis, and cancer [43]. Whole grains are associated with the morbidity and mortality of many diseases, which may be due in part to the impact on the intestinal flora [44]. In the present study, PHB supplementation significantly reversed the structure and composition of the gut microbiome in HFD mice to a near-normal status (Figure 3F). This is consistent with the results of animal experiments and in vitro experiments related to WHB. WHB, or its extract β-glucan, can regulate the intestinal flora, remarkably improve the species abundance in the intestine, and raise the ratio of beneficial bacteria such as *Bifidobacterium*, *Desulfovibrio*, and *Fusicatenibacter* which could produce short-chain fatty acids that contribute to host health [36,45,46]. 

*Lactobacillus* and *Bifidobacterium* have the potential to prevent chronic inflammation and deterioration of insulin resistance and are recognized as beneficial bacteria [47,48,49]. *Lactobacillus* can inhibit the diet-mediated increase in intestinal mucosal permeability by modifying the intestinal microbiota, thereby reducing circulating lipopolysaccharides and inflammatory cytokines, including IL-1β and IL-8, and ultimately alleviating inflammation and pancreatic β-cell dysfunction [50,51]. We found that *Bifidobacterium*, *Ileibacterium*, *Lactobacillus*, and *norank_f_Muribaculaceae* were significantly enriched in the feces of PHB supplemented mice (Figure 4D) and that the intestinal barrier structure was also improved (Figure 2F,G). This is consistent with previous research results [52].

The abundance of *Lactobacillus* displayed a dose-response relationship with PHB, which may be related to β-glucan. The content of β-glucan in the diet increased with the increased amount of PHB (Table S1). β-glucan is an indigestible carbohydrate that can be fermented by bacteria in the large intestine and mainly produces short-chain fatty acids (SCFAs)—in particular, acetate, propionate, and butyrate [53]. Among these, acetate is the main SCFA final product of *Bifidobacterium* fermentation, as well as *Bacteroides*, *Lactobacillus*, etc. [54]. SCFAs can reduce the expression of peroxisome proliferator-activated receptor-γ (PPAR-γ), leading to the increased oxidative metabolism of the liver and adipose tissue, reduced body fat accumulation, and hepatic steatosis, as well as increased insulin sensitivity. Additionally, Spearman’s analysis results showed that the intestinal microbes for beneficial bacteria, including *Lactobacillus*, *Bifidobacterium*, *Ileibacterium*, and *norank_f_Muribaculaceae*, and SCFAs-producing bacteria, e.g., *Lachnospiraceae_UCG_001*, *norank_f_Erysipelotrichaceae*, had a negative association with postprandial blood glucose level (Figure 4G).

Several limitations of this study should be considered. First, this is an animal experiment, and therefore it is a challenge to translate the findings in this study to humans [55] and identify the effective dose and intake time for humans. Second, raw HB was used in this study while HB is usually consumed after processing. Heat treatment could affect the nutrient composition of cereals [56] and we cannot predict whether the cooked HB has a hypoglycemic effect. Third, it is possible there could be a species-specific effect of HB on the physiological and metabolic functions of mice. Additionally, our study showed no positive effect of Zangqing 2000 on lipid metabolism; this is not the case in other studies using Zangqing 320 [20,38] or other species of HB [22]. Lastly, although our study explored the possible mechanism of HB lowering blood glucose via intestinal flora, the effect mechanism of HB on the carbohydrate metabolism pathway and others still needs to discovered. 

## 5. Conclusions

In summary, the intake of highland barley with a milling degree of 10% had a positive effect on mice fed a high-fat diet; this includes significantly decreasing FBG and postprandial blood glucose, inhibiting lipid droplet accumulation in the liver, improving colon damage, and restoring the imbalance of intestinal flora, including reducing the relative abundance of several HFD-related taxa and increasing the relative abundance of beneficial bacteria such as *Lactobacillus* and *Bifidobacterium*. However, it had no significant effect on bodyweight, adipose tissue weight, and the level of lipids in plasma. Overall, our research answered the question of whether the physiological function of highland barley after partial milling treatment exists which could have a guiding effect on the development of HB products.

## Data Availability

Not applicable.

## Supplementary Materials

The following are available online at https://www.mdpi.com/article/10.3390/nu14040762/s1, Table S1: Composition and energy supply of experimental diets, Table S2: Contents of the major nutrients and functional components of highland barley with different milling degrees, Table S3: Starch digestion properties in highland barley with different milling degrees, Table S4: eGI in highland barley with different milling degrees.

## References

[1] Wang W., Hu M., Liu H., Zhang X., Li H., Zhou F., Liu Y., Lei F., Qin J., Zhao Y. (2021). Global Burden of Disease Study 2019 suggests that metabolic risk factors are the leading drivers of the burden of ischemic heart disease. Cell Metab..

[2] Feigin V., Stark B., Johnson C., Roth G., Bisignano C., Abady G., Abbasifard M., Abbasi-Kangevari M., Abd-Allah F., Abedi V. (2021). Global, regional, and national burden of stroke and its risk factors, 1990–2019: A systematic analysis for the Global Burden of Disease Study 2019. Lancet Neurol..

[3] Schwingshackl L., Schwedhelm C., Hoffmann G., Knüppel S., Iqbal K., Andriolo V., Bechthold A., Schlesinger S., Boeing H. (2017). Food Groups and Risk of Hypertension: A Systematic Review and Dose-Response Meta-Analysis of Prospective Studies. Adv. Nutr..

[4] Wang W., Li J., Chen X., Yu M., Pan Q., Guo L. (2020). Whole grain food diet slightly reduces cardiovascular risks in obese/overweight adults: A systematic review and meta-analysis. BMC Cardiovasc. Disord..

[5] Wang Y., Duan Y., Zhu L., Fang Z., He L., Ai D., Jin Y. (2019). Whole grain and cereal fiber intake and the risk of type 2 diabetes: A meta-analysis. Int. J. Mol. Epidemiol. Genet..

[6] Hu Y., Ding M., Sampson L., Willett W.C., Manson J.E., Wang M., Rosner B., Hu F.B., Sun Q. (2020). Intake of whole grain foods and risk of type 2 diabetes: Results from three prospective cohort studies. BMJ.

[7] Schwingshackl L., Schwedhelm C., Hoffmann G., Knüppel S., Laure Preterre A., Iqbal K., Bechthold A., De Henauw S., Michels N., Devleesschauwer B. (2018). Food groups and risk of colorectal cancer. Int. J. Cancer.

[8] Zhang X.F., Wang X.K., Tang Y.J., Guan X.X., Guo Y., Fan J.M., Cui L.L. (2020). Association of whole grains intake and the risk of digestive tract cancer: A systematic review and meta-analysis. Nutr. J..

[9] Ievina S., Arta K., Vija S., Aina K., Mauritz A., Kari B.O.A., Vita S., Evita S. (2018). Adaptability of hull-less barley varieties to different cropping systems and climatic conditions. Acta Agric. Scand. Sect. B-Soil Plant Sci..

[10] Sterna V., Zute S., Jansone I., Kantane I. (2017). Chemical Composition of Covered and Naked Spring Barley Varieties and Their Potential for Food Production. Pol. J. Food Nutr. Sci..

[11] Zhang G., Junmei W., Jinxin C. (2002). Analysis of β-glucan content in barley cultivars from different locations of China. Food Chem..

[12] Obadi M., Sun J., Xu B. (2021). Highland barley: Chemical composition, bioactive compounds, health effects, and applications. Food Res. Int..

[13] Hu J., Wu Y., Xie H., Shi W., Chen Z., Jiang D., Hu H., Zheng X., Xu J., Yang Y. (2020). Purification, Preliminary Structural Characterization, and In Vitro Inhibitory Effect on Digestive Enzymes by beta-Glucan from Qingke (Tibetan Hulless Barley). Adv. Polym. Tech..

[14] Liu Z., Li B. (2021). Chlorogenic acid and beta-glucan from highland barley grain ameliorate beta-cell dysfunction via inhibiting apoptosis and improving cell proliferation. Food Funct..

[15] Liu Z., Li B. (2021). (-)-Epicatechin and beta-glucan from highland barley grain modulated glucose metabolism and showed synergistic effect via Akt pathway. J. Funct. Foods.

[16] Cheng D., Zhang X., Meng M., Han L., Li C., Hou L., Qi W., Wang C. (2016). Inhibitory effect on HT-29 colon cancer cells of a water-soluble polysaccharide obtained from highland barley. Int. J. Biol. Macromol..

[17] Liao Z., Cai H., Xu e., Wang J., Qiu C., Xie J., Huang W., Sui Z. (2018). Protective Role of Antioxidant Huskless Barley Extracts on TNF-α-Induced Endothelial Dysfunction in Human Vascular Endothelial Cells. Oxid. Med. Cell. Longev..

[18] Liu L., Wang X., Li Y., Sun C. (2015). Postprandial Differences in the Amino Acid and Biogenic Amines Profiles of Impaired Fasting Glucose Individuals after Intake of Highland Barley. Nutrients.

[19] Gong L., Gong L., Zhang Y. (2014). Intake of Tibetan hull-less barley is associated with a reduced risk of metabolic related syndrome in rats fed high-fat-sucrose diets. Nutrients.

[20] Deng N., Guo R., Zheng B., Li T., Liu R.H. (2020). IRS-1/PI3K/Akt pathway and miRNAs are involved in whole grain highland barley (Hordeum vulgare L.) ameliorating hyperglycemia of db/db mice. Food Funct..

[21] Deng N., He Z., Guo R., Zheng B., Li T., Liu R.H. (2020). Highland Barley Whole Grain (Hordeum vulgare L.) Ameliorates Hyperlipidemia by Modulating Cecal Microbiota, miRNAs, and AMPK Pathways in Leptin Receptor-Deficient db/db Mice. J. Agric. Food Chem..

[22] Guo T., Horvath C., Chen L., Chen J., Zheng B. (2020). Understanding the nutrient composition and nutritional functions of highland barley (Qingke): A review. Trends Food Sci. Technol..

[23] Lian Q., Cui M., Li J., Jia P., Xiao M. (2021). Analysis on factors affecting mycotoxin production in highland barley raw grains. Food Safe Qual. Detec. Technol..

[24] Wei N., Yue X., Yu Q., Zhang F. (2020). Study on mycotoxin contamination and toxigenic fungi pollution of main crops in Tibet plateau. J. Triticeae Crops.

[25] Barrett E.M., Batterham M.J., Ray S., Beck E.J. (2019). Whole grain, bran and cereal fibre consumption and CVD: A systematic review. Br. J. Nutr..

[26] Aune D., Norat T., Romundstad P., Vatten L.J. (2013). Whole grain and refined grain consumption and the risk of type 2 diabetes: A systematic review and dose-response meta-analysis of cohort studies. Eur. J. Epidemiol..

[27] Liu X., Lai H., Mi B., Qi X., Gan W., Du H. (2020). Associations of Coarse Grain Intake with Undiagnosed Hypertension among Chinese Adults: Results from the China Kadoorie Biobank. Nutrients.

[28] Miller K.B. (2020). Review of whole grain and dietary fiber recommendations and intake levels in different countries. Nutr. Rev..

[29] Tan B., Wu N., Zhai X. (2020). Solutions for whole grain food development. Nutr. Rev..

[30] McMackin E., Dean M., Woodside J.V., McKinley M.C. (2013). Whole grains and health: Attitudes to whole grains against a prevailing background of increased marketing and promotion. Public Health Nutr..

[31] Jones J.M., Adams J., Harriman C., Miller C., Van Der Kamp J.W. (2015). Nutritional Impacts of Different Whole Grain Milling Techniques: A Review of Milling Practices and Existing Data. Cereal Foods World.

[32] Obadi M., Qi Y., Xu B. (2021). Highland barley starch (Qingke): Structures, properties, modifications, and applications. Int. J. Biol. Macromol..

[33] Thielecke F., Jonnalagadda S.S. (2014). Can Whole Grain Help in Weight Management?. J. Clin. Gastroenterol..

[34] Liu S.M., Willett W.C., Manson J.E., Hu F.B., Rosner B., Colditz G. (2003). Relation between changes in intakes of dietary fiber and grain products and changes in weight and development of obesity among middle-aged women. Am. J. Clin. Nutr..

[35] Sadeghi O., Sadeghian M., Rahmani S., Maleki V., Larijani B., Esmaillzadeh A. (2020). Whole-Grain Consumption Does Not Affect Obesity Measures: An Updated Systematic Review and Meta-analysis of Randomized Clinical Trials. Adv. Nutr..

[36] Zheng B., Zhong S., Tang Y., Chen L. (2020). Understanding the nutritional functions of thermally-processed whole grain highland barley in vitro and in vivo. Food Chem..

[37] Xia X., Li G., Song J., Zheng J., Kan J. (2018). Hypocholesterolaemic effect of whole-grain highland hull-less barley in rats fed a high-fat diet. Br. J. Nutr..

[38] Xia X., Li G., Ding Y., Ren T., Zheng J., Kan J. (2017). Effect of Whole Grain Qingke (Tibetan Hordeum vulgare L. Zangqing 320) on the Serum Lipid Levels and Intestinal Microbiota of Rats under High-Fat Diet. J. Agric. Food Chem..

[39] Guo H., Lin S., Lu M., Gong J.D.B., Wang L., Zhang Q., Lin D.R., Qin W., Wu D.T. (2018). Characterization, in vitro binding properties, and inhibitory activity on pancreatic lipase of beta-glucans from different Qingke (Tibetan hulless barley) cultivars. Int. J. Biol. Macromol..

[40] Yang X.J., Dang B., Fan M.T. (2018). Free and Bound Phenolic Compound Content and Antioxidant Activity of Different Cultivated Blue Highland Barley Varieties from the Qinghai-Tibet Plateau. Molecules.

[41] Deng N., Zheng B., Li T., Liu R.H. (2020). Assessment of the Phenolic Profiles, Hypoglycemic Activity, and Molecular Mechanism of Different Highland Barley (Hordeum vulgare L.) Varieties. Int. J. Mol. Sci..

[42] Liu Z.H., Li B. (2021). Procyanidin B1 and p-Coumaric Acid from Highland Barley Grain Showed Synergistic Effect on Modulating Glucose Metabolism via IRS-1/PI3K/Akt Pathway. Mol. Nutr. Food Res..

[43] Zhang Y.J., Li S., Gan R.Y., Zhou T., Xu D.P., Li H.B. (2015). Impacts of Gut Bacteria on Human Health and Diseases. Int. J. Mol. Sci..

[44] Seal C.J., Courtin C.M., Venema K., de Vries J. (2021). Health benefits of whole grain: Effects on dietary carbohydrate quality, the gut microbiome, and consequences of processing. Compr. Rev. Food Sci. Food Saf..

[45] Gong L., Cao W., Gao J., Wang J., Zhang H., Sun B., Yin M. (2018). Whole Tibetan Hull-Less Barley Exhibit Stronger Effect on Promoting Growth of Genus Bifidobacterium than Refined Barley In Vitro. J. Food Sci..

[46] Nie C., Yan X., Xie X., Zhang Z., Zhu J., Wang Y., Wang X., Xu N., Luo Y., Sa Z. (2021). Structure of beta-glucan from Tibetan hull-less barley and its in vitro fermentation by human gut microbiota. Chem. Biol. Technol. Agric..

[47] Soleimani A., Zarrati Mojarrad M., Bahmani F., Taghizadeh M., Ramezani M., Tajabadi-Ebrahimi M., Jafari P., Esmaillzadeh A., Asemi Z. (2017). Probiotic supplementation in diabetic hemodialysis patients has beneficial metabolic effects. Kidney Int..

[48] Toshimitsu T., Gotou A., Sashihara T., Hachimura S., Shioya N., Suzuki S., Asami Y. (2020). Effects of 12-Week Ingestion of Yogurt Containing Lactobacillus plantarum OLL2712 on Glucose Metabolism and Chronic Inflammation in Prediabetic Adults: A Randomized Placebo-Controlled Trial. Nutrients.

[49] Tiderencel K.A., Hutcheon D.A., Ziegler J. (2020). Probiotics for the treatment of type 2 diabetes: A review of randomized controlled trials. Diabetes Metab. Res. Rev..

[50] Sakai Y., Arie H., Ni Y., Zhuge F., Xu L., Chen G., Nagata N., Suzuki T., Kaneko S., Ota T. (2020). Lactobacillus pentosus strain S-PT84 improves steatohepatitis by maintaining gut permeability. J. Endocrinol..

[51] Tian P., Li B., He C., Song W., Hou A., Tian S., Meng X., Li K., Shan Y. (2016). Antidiabetic (type 2) effects of Lactobacillus G15 and Q14 in rats through regulation of intestinal permeability and microbiota. Food Funct..

[52] Fernandez-Julia P.J., Munoz-Munoz J., van Sinderen D. (2021). A comprehensive review on the impact of β-glucan metabolism by Bacteroides and Bifidobacterium species as members of the gut microbiota. Int. J. Biol. Macromol..

[53] Cummings J.H., Pomare E.W., Branch W.J., Naylor C., Macfarlane G.T. (1987). Short chain fatty acids in human large intestine, portal, hepatic and venous blood. Gut.

[54] Wang H., Ren P., Mang L., Shen N., Chen J., Zhang Y. (2019). In vitro fermentation of novel microwave-synthesized non-digestible oligosaccharides and their impact on the composition and metabolites of human gut microbiota. J. Funct. Foods.

[55] Brunkwall L., Orho-Melander M. (2017). The gut microbiome as a target for prevention and treatment of hyperglycaemia in type 2 diabetes: From current human evidence to future possibilities. Diabetologia.

[56] Zhao Q., Hou D., Laraib Y., Xue Y., Shen Q. (2021). Comparison of the effects of raw and cooked adzuki bean on glucose/lipid metabolism and liver function in diabetic mice. Cereal Chem..

