# Supplementary material for: Beneficial Effects of Partly Milled Highland Barley on the Prevention of High-Fat Diet-Induced Glycometabolic Disorder and the Modulation of Gut Microbiota in Mice
Source: Nutrients. 2022 Feb 11;14(4):762. doi: 10.3390/nu14040762 (PMC8877997; doi:10.3390/nu14040762)
Supplement: Supplementary file 1 [file nutrients-14-00762-s001.zip › nutrients-1551307-supplementary.pdf]

Table S1. Composition and energy supply of experimental diets

Ingredient (g/kg)	NC [1]	MC	LD	MD	HD
PHB flour, 60 mesh	0	0	100	200	300
Casein	200	258.46	247.07	235.66	224.27
L-Cystine	3	3.88	3.88	3.88	3.88
Corn Starch	506.2	0	0	0	0
Maltodextrin 10	125	161.54	89.22	16.82	0
Sucrose	68.8	88.91	88.91	88.91	33.42
Cellulose, BW200	50	64.62	51.75	38.86	25.99
Soybean Oil	25	32.31	28.89	25.47	22.06
Lard	20	316.61	316.60	316.60	316.60
Mineral Mix S10026	10	12.92	12.92	12.92	12.92
DiCalcium Phosphate	13	16.80	16.80	16.80	16.80
Calcium Carbonate	5.5	7.11	7.11	7.11	7.11
Potassium Citrate, 1 H2O	16.5	21.32	21.32	21.32	21.32
Vitamin Mix, V10001	10	12.92	12.92	12.92	12.92
Choline Bitartrate	2	2.58	2.58	2.58	2.58
FD&C Blue Dye #1	0.01	0.06	0	0	0
FD&C Yellow Dye #5	0.04	0	0	0	0
Total (g)	1000.00	1000.00	999.97	999.85	999.87
Calculated energy (kcal%)					
Protein	20	20	20	20	20
Fat	10	60	60	60	60
Carbohydrate	70	20	20	20	20
β-glucan (g/100g)	- [2]	-	0.45	0.85	1.27

[1] Abbreviations: PHB, partly milled highland barley; NC, normal control group fed a low-fat diet; MC, model control group fed a high-fat diet (HFD); LD, low-dose group fed HFD containing 10% PHB; MD, middle-dose group fed HFD containing 20% PHB; HD, high-dose group fed HFD containing 30% PHB.

[2] "-": Not detected.

Table S2. Contents of the major nutrients and functional components of highland barley with different milling degrees

Milling degree (%)	Starch (g/100g)	Protein (g/100g)	Fat (g/100g)	Total fiber (g/100g)	β-glucan (g/100g)	Total polyphenolics (mg/kg)
10	62.5	11.1	4.5	8.9	4.47	4305.0
20	63.8	9.9	2.7	6.6	3.39	3571.0
30	67.5	8.8	1.1	5.5	4.52	3158.7

Table S3. Starch digestion properties in highland barley with different milling degrees

Milling degree	Starch		
(%)	RDS (%)	SDS (%)	RS (%)
10	33.28	44.99	21.73
20	37.09	43.29	19.62
30	40.99	39.11	19.90

Abbreviations: RDS, Rapidly Digestible Starch; SDS, Slowly Digestible Starch; RS, Resistant Starch.

Table S4. eGI in higland barley with different milling degrees

Milling degree (%)	C∞	k	eGI (Bread = 100)
10	82.46	0.018	77.02
20	82.98	0.021	83.54
30	83.29	0.022	88.28

Note: C∞ and k represent the maximum hydrolysis extent and the kinetic constant, respectively; eGI, Estimated Glycemic Index.
